# Comparison of the Diagnostic Performances of Ultrasound-Based Models for Predicting Malignancy in Patients With Adnexal Masses

**DOI:** 10.3389/fonc.2021.673722

**Published:** 2021-06-01

**Authors:** Le Qian, Qinwen Du, Meijiao Jiang, Fei Yuan, Hui Chen, Weiwei Feng

**Affiliations:** ^1^ Department of Obstetrics and Gynecology, Ruijin Hospital, Shanghai Jiaotong University School of Medicine, Shanghai, China; ^2^ Department of Pathology, Ruijin Hospital, Shanghai Jiaotong University School of Medicine, Shanghai, China

**Keywords:** adnexal mass, tumor, diagnosis, ultrasonography, prediction model

## Abstract

**Aim:**

This study aimed to compare different ultrasound-based International Ovarian Tumor Analysis (IOTA) prediction models, namely, the Simple Rules (SRs) the Assessment of Different NEoplasias in the adneXa (ADNEX) models, and the Risk of Malignancy Index (RMI), for the pre-operative diagnosis of adnexal mass.

**Methods:**

This single-centre diagnostic accuracy study involved 486 patients. All ultrasound examinations were analyzed and the prediction models were applied. Pathology was the clinical reference standard. The diagnostic performances of prediction models were measured by evaluating receiver-operating characteristic curves, sensitivities, specificities, positive and negative predictive values, positive and negative likelihood ratios, and diagnostic odds ratios.

**Results:**

To discriminate benign and malignant tumors, areas under the ROC curves (AUCs) for ADNEX models were 0.94 (95% CI: 0.92–0.96) with CA125 and 0.94 (95% CI: 0.91–0.96) without CA125, which were significantly higher than the AUCs for RMI I-III: 0.87 (95% CI: 0.83–0.90), 0.83 (95% CI: 0.80–0.86), and 0.82 (95% CI: 0.78–0.86), (all P < 0.0001). At a cut-off of 10%, the ADNEX model with CA125 had the highest sensitivity (0.93; 95% CI: 0.87–0.97) compared with the other models. The SRs model achieved a sensitivity of 0.93 (95% CI: 0.86–0.97) and a specificity of 0.86 (95% CI: 0.82–0.89) when inconclusive diagnoses (11.7%) were classified as malignant.

**Conclusion:**

ADNEX and SRs models were excellent at characterising adnexal masses which were superior to the RMI in Chinese patients.

## Introduction

Ovarian cancer (OC) has the highest mortality rate and most unfavourable prognosis among the gynaecological malignancies; the average 5-year survival rate is < 50% (1, 2). Currently, transvaginal ultrasound is the most commonly used, non-invasive, affordable imaging technique for pre-operative evaluations of adnexal masses with minimal risk and discomfort to the patient (3–5). And subjective assessments of ultrasound findings by specialists in gynaecological ultrasonography are one of the best means of evaluating adnexal masses in clinical practice (5–10). To optimise the treatment and improve the survival of patients with malignant ovarian tumors, and to avoid unnecessary interventions in and preserve the fertility of patients with benign ovarian tumors, accurately characterising benign and malignant ovarian masses through appropriate staging is essential (11, 12). In particularly, accurate diagnosis of borderline ovarian tumors (BOTs) is critical to ensure timely and appropriate management, especially in women desiring to preserve fertility (13–17).

Several ultrasound-based prediction models have been developed to accurately discriminate between benign and malignant tumors, because the numbers of experienced examiners are insufficient and they are unavailable in some regions (18). The Risk of Malignancy Index (RMI), which accounts for the serum cancer antigen (CA) 125 levels, menopausal status, and the ultrasound findings, is a prediction model that is recommended by many national guidelines (19–21). However, the procedures used to calculate the RMI are time-consuming, and its diagnostic performance is unsatisfactory. The International Ovarian Tumor Analysis (IOTA) group presented a consensus statement about the ultrasound characteristics of adnexal tumors in 2000 (22), and other diagnostic models were subsequently developed and validated, including the Logistic Regression model 2 (23, 24), simple ultrasound-based rules or Simple Rules (SRs) model (25–27), and the Assessment of Different NEoplasias in the adneXa (ADNEX) model (28). The findings from previous external validation studies have shown that the SRs model is easy to use and its diagnostic performance is good, but it is not suitable for all adnexal masses (25–27). Although the ADNEX model is excellent at differentiating between malignant and benign tumors (6, 29–31) and indicating the stages of malignant tumors, there is still no diagnostic accuracy study to compare these models above-mentioned in a Chinese setting.

This study aimed to compare the ADNEX and SRs models, and the RMI regarding their abilities to discriminate between benign and malignant adnexal masses using data from a single oncology centre in China.

## Material and Methods

### Study Setting and Design

Between June 2017 and June 2018, the study was carried out using data prospectively collected from consecutive patients. It evaluated the diagnostic performances of the ADNEX and SRs models, and variants of the RMI (I–III) within a population of women who underwent surgery to remove adnexal masses at the Department of Obstetrics and Gynaecology in a tertiary referral oncology centre. All of the patients underwent pre-operative transvaginal or transrectal ultrasonography examinations according to the IOTA protocol (22) to assess the morphology of the adnexal masses. Clinicians made the final decisions regarding surgery and clinical judgments.

### Participants

The patients were prospectively and consecutively enrolled, and they presented with ≥ 1 ultrasound-diagnosed adnexal mass. The inclusion criteria were ≥ 1 adnexal mass detected by transvaginal or transrectal ultrasonography that was not a physiological cyst, patients who were prepared to undergo surgery based on a clinician’s recommendation, and a time interval of 30 days between ultrasound and surgery.

Participants were excluded from the study if they failed to undergo surgery, they were diagnosed with a recurrence of OC, they had undergone a bilateral adnexectomy previously, they had an ectopic pregnancy, or their clinical data were incomplete. A total of 486 patients were included in the final analysis. The study was approved by the Ruijin Hospital, Shanghai Jiaotong University School of Medicine institutional ethics (Grant No.2018-136).

### Data Collection

All patients underwent pre-operative transvaginal or transrectal ultrasonography using Voluson E10 (GE Healthcare) and iU22 (Philips Healthcare) ultrasound machines with 5.0–9.0 MHz and 4.0–8.0 MHz transvaginal probes, and 1.0–5.0 MHz transabdominal probes, and the findings were recorded. When a malignancy was suspected or a mass was too large to be evaluated using transvaginal ultrasonography alone, transabdominal ultrasonography was performed. Two expert ultrasonographers with ≥ 10 years of experience in gynaecological ultrasound assessed the tumors’ pre-operative sonographic morphologies using the IOTA protocol’s nomenclature and methodology (22). After the ultrasound examinations and before the statistical analysis of the data, we applied the ADNEX model and three variants of the RMI to calculate the risk of malignancy without knowledge of the histological findings. When multiple adnexal masses were detected, we analysed the mass with the most complex ultrasonographic morphology, and when masses had similar morphological characteristics, we chose the largest mass (22, 28).

Before the ultrasound examinations, we collected clinical data describing the patients’ ages, menopausal statuses, previous malignancies, and family histories of OC. The patients’ pre-operative CA125 levels were measured using a chemoluminescence technique and an automatic analyser (i2000SN; Abbott AxSYM).

### Prediction Models

Three prediction models were used to differentiate between benign and malignant adnexal masses. The ADNEX model is available at no cost on the IOTA website (https://www.iotagroup.org/iota-models-software/adnex-risk-model) or it can be installed as a mobile phone application; it comprises nine predictors, including three clinical and six ultrasound variables (28). After inputting all the predictors objectively, the probability ratios for a benign or a malignant mass are displayed both graphically and numerically. As it is the first multiclass prediction model for adnexal masses, the likelihoods of a mass being a BOT, stage I OC, stages II–IV OC, or a metastasis are presented. The ADNEX model is available in versions that include and exclude the CA125 level, and we evaluated the predictive accuracy of the ADNEX model with and without CA125 in this study.

The SRs model comprises a set of rules based on five ultrasound features that indicate benignity (B-features) and five features that indicate malignancy (M-features) (25–27). A lesion is classified as benign if ≥ 1 B-feature is present in the absence of any M-features, and malignant if ≥ 1 M-feature is present in the absence of any B-features. If both B-features and M-features are present or if none of the features are present, the model yields an inconclusive result.

Three principal variants of the RMI scoring system (RMI-I, RMI-II, and RMI-III) were applied that combined the ultrasound findings, serum CA125 levels, and menopausal status (19–21). The points attributed to patients’ ultrasound findings and menopausal statuses differ for the RMI variants, and these points generate a score; a total score of ≥ 200 points was used as the cut-off for malignancy.

### Reference Standard

Pathology was the reference standard used for all patients in this study. Tissue specimens obtained during surgery were analysed by a team of pathologists who specialised in gynaecological pathology and were unaware of the ultrasound findings. The tumors were classified according to the World Health Organization’s guidelines for the classification of tumors (32). The stages of the malignant tumors were defined using the International Federation of Gynecology and Obstetrics 2012 criteria (33).

### Statistical Analyses

Basic discrimination between benign and malignant adnexal masses by the ADNEX model with or without the CA125 levels and the three RMI variants was assessed using receiver-operating characteristic curves (ROCs) and summarised by calculating the areas under the curves (AUCs). The prediction methods’ AUCs were compared using the method described by DeLong et al. (34). As AUCs could not be calculated for the SRs model, which is based on categorical variables, the McNemar test was used to assess the model’s discrimination between benign and malignant adnexal masses. Diagnostic performance measures, including the sensitivities, specificities, positive and negative predictive values, positive and negative likelihood ratios, and the diagnostic odds ratios (DORs), were calculated to evaluate the models’ classifications of benign or malignant tumors using cut-off points proposed in previous publications (6, 19–21, 28).

The ultrasonographic and clinical characteristics of, and the CA125 levels associated with the benign and malignant tumors were compared; the chi-square test and Fisher’s exact test were used to analyse the categorical data, and the Mann-Whitney U-test was used to analyse the continuous data. The statistical analyses were conducted using IBM^®^SPSS^®^ software, version 22.0 (IBM Corporation) and MedCalc Statistical Software, version 15.2.2 (MedCalc Software bvba). BOTs were considered malignant for the purposes of the statistical analyses. All of the statistical calculations were performed using 95% confidence intervals (CIs), and a value of P < 0.05 was considered statistically significant.

## Results

### Clinical Findings and Pathologic Diagnosis

**Figure 1:**
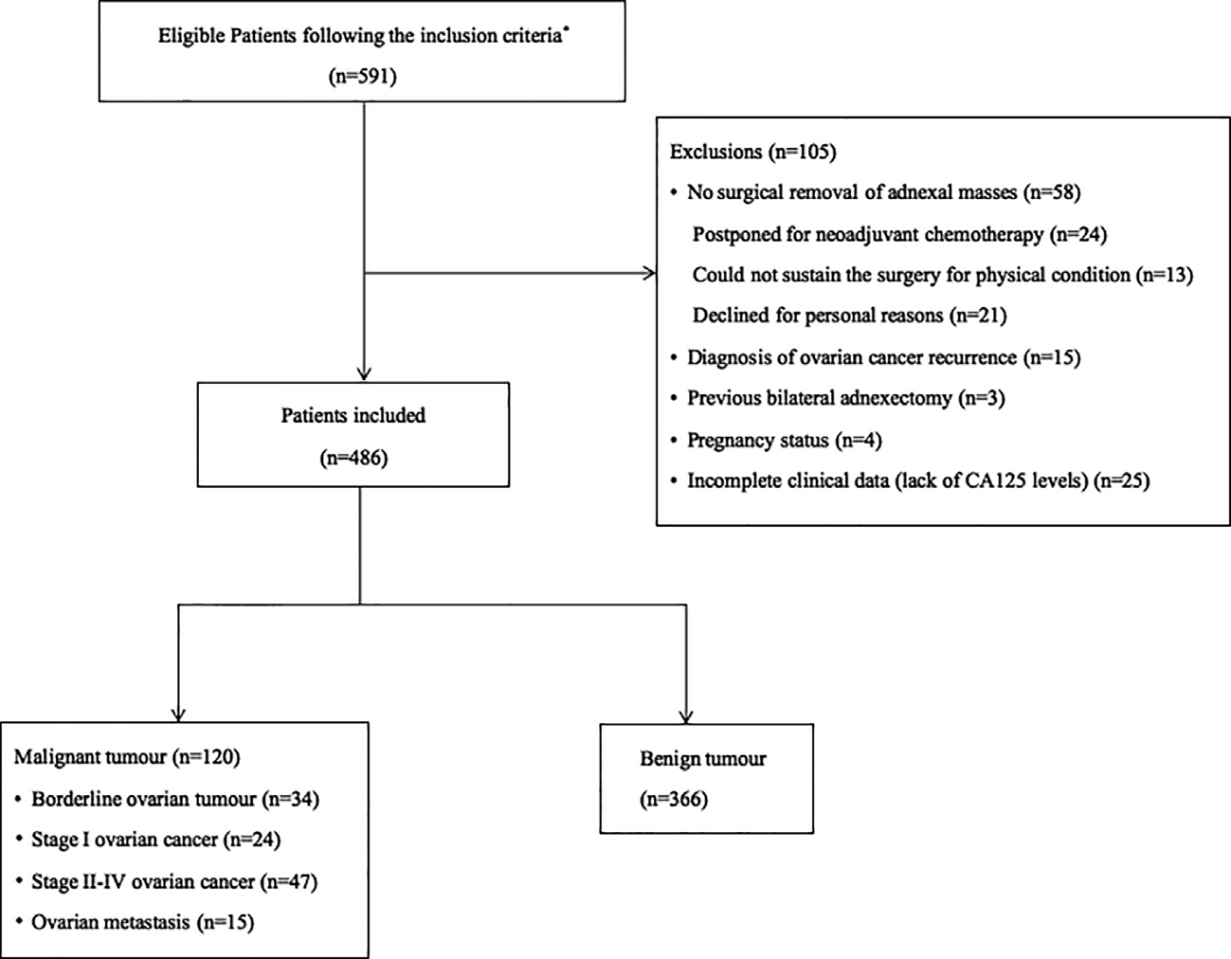
Flow diagram summarizing the inclusion of patients with adnexal masses in the study. *The inclusion criteria were as follows: (1) patients with at least one adnexal mass detected by transvaginal or transrectal ultrasonography, (2) patients who were prepared to undergo surgery, (3) patients with a time interval between ultrasound and surgery within 30 days, and (4) patients without a previous history of ovarian cancer.

Between June 2017 and June 2018, 591 consecutive women with adnexal tumors who underwent pre-operative ultrasound examinations were prospectively enrolled. The final cohort consisted of 486 women; 105 women met the exclusion criteria and were excluded from study. **Figure 1** provides a detailed overview of the patients’ inclusion and exclusion from the study.

In the final analysis, 486 patients with 366 (75.3%) benign and 120 (24.7%) malignant adnexal masses were included. **Table 1** presents the histological results. Endometriomas (19.8%, 96/486) and serous cystadenomas (13.4%, 65/486) were the most common benign diagnoses. Among the malignant masses, 7.0% (34/486) were BOTs, 4.9% (24/486) were stage I OCs, 9.7% (47/486) were stages II–IV OCs, and 3.1% (15/486) were metastases.

**Table 1:** Distributions of histology outcomes of 486 adnexal masses.

Histological type of masses	N (%)
Benign	366 (75.3)
Endometrioma	96 (19.8)
Serous cystadenoma	65 (13.4)
Teratoma	46 (9.5)
Mucinous cystadenoma	34 (7.0)
Hydrosalpinx	32 (6.6)
Fibrothecoma	20 (4.1)
Mesosalpinx cyst	17 (3.5)
Parovarian cyst	14 (2.9)
Cystadenofibroma	6 (1.2)
Fibroma	6 (1.2)
Adenofibroma	4 (0.8)
Brenner tumor	4 (0.8)
Peritoneal mesothelioma	3 (0.6)
Sertoli-Leydig cell tumor	2 (0.4)
Sclerosing stromal tumor	1 (0.2)
Tuberculosis	1 (0.2)
Other ovarian benign lesion	15 (3.1)
Borderline	34 (7.0)
Serous	16 (3.3)
Mucinous	15 (3.1)
Endometrioid	3 (0.6)
Primary ovarian malignant	71(14.6)
Serous adenocarcinoma	42 (8.6)
Clear cell carcinoma	10 (2.1)
Endometrioid adenocarcinoma	6 (1.2)
Mucinous adenocarcinoma	3 (0.6)
Sertoli-Leydig cell tumor	2 (0.4)
Carcinosarcoma	2 (0.4)
Granulosa cell tumor	2 (0.4)
Seromucinous adenocarcinoma	1 (0.2)
Diffuse large B cell lymphoma of ovary	1 (0.2)
Small cell neuroendocrine carcinoma	1 (0.2)
Strumal carcinoid of ovary	1 (0.2)
Metastasis	15 (3.1)
Gastric cancer	6 (1.2)
Appendiceal adenocarcinoma	3 (0.6)
Cholangiocarcinoma	2 (0.4)
Breast cancer	2 (0.4)
Pancreatic cancer	2 (0.4)

**Table 2** summarises the patients’ clinical characteristics and data describing the ultrasound findings from the benign and malignant tumors. The patients with malignancies were older, were more likely to be post-menopausal and to have a family history of OC, and had higher CA125 levels than those with benign tumors (all P < 0.05). Regarding the ultrasound findings, the malignant tumors had significantly greater diameters, more solid tissue, wider solid tissue components, > 10-cyst locules, more papillary projections, and more ascites compared with the benign masses (all P < 0.001). None of the patients with malignant tumors had acoustic shadows.

**Table 2:** Results regarding clinical characteristics and ultrasound features for 486 patients with adnexal mass.

Characteristic	Benign(n = 366)	Malignant(n = 120)	P
Age (years)	41 (31-51)	54 (42-63)	<0.001*
Menopausal status			<0.001**
Premenopausal	275 (75.1)	53 (44.2)	
Postmenopausal	91 (24.9)	67 (55.8)	
CA125 (U/mL)	10 (11-38)	54 (18-517)	<0.001*
Family history of OC	0 (0.0)	2 (1.7)	0.013***
Maximal diameter of lesion (mm)	56 (44-72)	78 (50-126)	<0.001*
Presence of solid tissue	108 (29.5)	107 (89.2)	<0.001**
Proportion solid tissue if present (mm)	31 (17-46)	44 (20-65)	<0.001*
Presence of papillary projections	39 (10.7)	46 (38.3)	<0.001**
0	327 (89.3)	74 (61.7)	
1	21 (5.7)	13 (10.8)	
2	6 (1.6)	3 (2.5)	
3	5 (1.4)	5 (4.2)	
>3	7 (1.9)	25 (20.8)	
>10-cyst locules	11 (3.0)	22 (18.3)	<0.001**
Acoustic shadows	43 (11.7)	0 (0.0)	<0.001***
Ascites	2 (0.5)	35 (29.2)	<0.001**

Data are given as n (%) for categorical data and median (interquartile range) for continuous data.

*Mann-Whitney U-test for continuous data, **Chi-square test and ***Fisher’s exact test for categorical data. OC, ovarian cancer.

### Diagnostic Performance of Adnexal Mass Prediction Models

**Table 3** details the diagnostic performances of the adnexal mass prediction models regarding their discrimination between benign and malignant tumors. The AUCs for the ADNEX models for differentiating malignant tumors from benign tumors that did and did not account for the CA125 level were 0.94 (95% CI: 0.92–0.96) and 0.94 (95% CI: 0.91–0.96), respectively. At a cut-off of 10%, the performance of the prediction model that included CA125 was excellent, with a sensitivity of 0.93 (95% CI: 0.87–0.97), a specificity of 0.76 (95% CI: 0.72–0.81), and a DOR of 43.67, and the performance of the prediction model that did not include CA125 had a sensitivity of 0.93 (95% CI: 0.87–0.97), a specificity of 0.74 (95% CI: 0.69–0.79), and a DOR of 40.00.

**Table 3:** Diagnostic performance of the prediction models for discrimination between benign and malignant adnexal masses.

Assessment method	AUC	Sensitivity	Specificity	PPV	NPV	LR+	LR-	DOR
ADNEX^125^	0.94 (0.92-0.96)	0.93 (0.87-0.97)	0.76 (0.72-0.81)	0.80 (0.75-0.84)	0.92 (0.87-0.95)	3.93 (3.20-4.72)	0.09 (0.04-0.22)	43.67
ADNEX^N125^	0.94 (0.91-0.96)	0.93 (0.87-0.97)	0.74 (0.69-0.79)	0.78 (0.73-0.83)	0.92 (0.87-0.95)	3.60 (3.00-4.31)	0.09 (0.05-0.20)	40.00
SRs+BE	NA	0.69 (0.60-0.77)	0.96 (0.93-0.97)	0.94 (0.90-0.97)	0.76 (0.70-0.80)	15.82 (9.66-25.93)	0.32 (0.25-0.42)	49.44
SRs+MAL	NA	0.93 (0.86-0.97)	0.86 (0.82-0.89)	0.87 (0.82-0.91)	0.92 (0.88-0.95)	6.51 (5.04-8.42)	0.09 (0.05-0.16)	72.33
RMI-I	0.87 (0.83-0.90)	0.55 (0.46-0.64)	0.93 (0.90-0.96)	0.89 (0.83-0.94)	0.67 (0.62-0.72)	8.05 (5.33-12.19)	0.48 (0.43-0.59)	16.77
RMI-II	0.83 (0.80-0.86)	0.61 (0.52-0.70)	0.92 (0.89-0.95)	0.89 (0.83-0.93)	0.70 (0.65-0.75)	7.95 (5.42-11.75)	0.42 (0.33-0.52)	18.93
RMI-III	0.82 (0.78-0.86)	0.53 (0.44-0.63)	0.94 (0.91-0.96)	0.90 (0.84-0.95)	0.67 (0.62-0.72)	9.30 (5.91-14.49)	0.50 (0.45-0.63)	18.60

Values in parentheses are 95% CI. Prediction models: ADNEX^125^, the Assessment of Different NEoplasias in the adneXa model with CA125 level; ADNEX^N125^, the Assessment of Different NEoplasias in the adneXa model without CA125 level; SRs+BE, International Ovarian Tumor Analysis simple ultrasound-based rules applied with inconclusive tumors (13.2%, 64/486 cases) being classified as benign; SRs+MAL, International Ovarian Tumor Analysis simple ultrasound-based rules applied with inconclusive results being categorised as malignant; RMI-I, RMI-II, RMI-III, three variants of the Risk of Malignancy Index. For ADNEX models, cut-off value of 10% was used and for the three variants of RMI model, cut-off value of 200 was used. AUC, area under receiver-operating characteristic curve; PPV, positive predictive value; NPV, negative predictive value; LR+, positive likelihood ratio; LR–, negative likelihood ratio; DOR, diagnostic odds ratio; NA, not applicable.

The SRs model was applicable to 422 (86.8%) patients with adnexal tumors. Of the tumors with inconclusive diagnoses, 56.3% (36/64) were benign tumors, 20.3% (13/64) were BOTs, 14.1% (9/64) were stage I OCs, 4.7% (3/64) were stages I–IV OCs, 4.7% (3/64) were metastases, approximately 43.8% were malignant histologically, and most of the benign masses (75.0%, 27/36) presented with a solid component. When the masses with inconclusive diagnoses were classified as benign, the SRs model’s diagnostic performance had a sensitivity of 0.69 (95% CI: 0.60–0.77), a specificity of 0.96 (95% CI: 0.93–0.97), and a DOR of 49.44, and when they were categorised as malignant, the SRs model’s diagnostic performance had a sensitivity of 0.93 (95% CI: 0.86–0.97), a specificity of 0.86 (95% CI: 0.82–0.89), and a DOR of 72.33.

The three RMI variants with cut-offs of 200 showed poor diagnostic performances in relation to the adnexal masses. Regarding RMI variants I, II, and III, the AUCs for differentiating malignant tumors from benign tumors were 0.87 (95% CI: 0.83–0.90), 0.83 (95% CI: 0.80–0.86), and 0.82 (95% CI: 0.78–0.86), respectively, with sensitivities of 0.55 (95% CI: 0.46–0.64), 0.61 (95% CI: 0.52–0.70), and 0.53 (95% CI: 0.44–0.63), respectively, and specificities of 0.93 (95% CI: 0.90–0.96), 0.92 (95% CI: 0.89–0.95), and 0. 94 (95% CI: 0.91–0.96), respectively.

**Figure 2:**
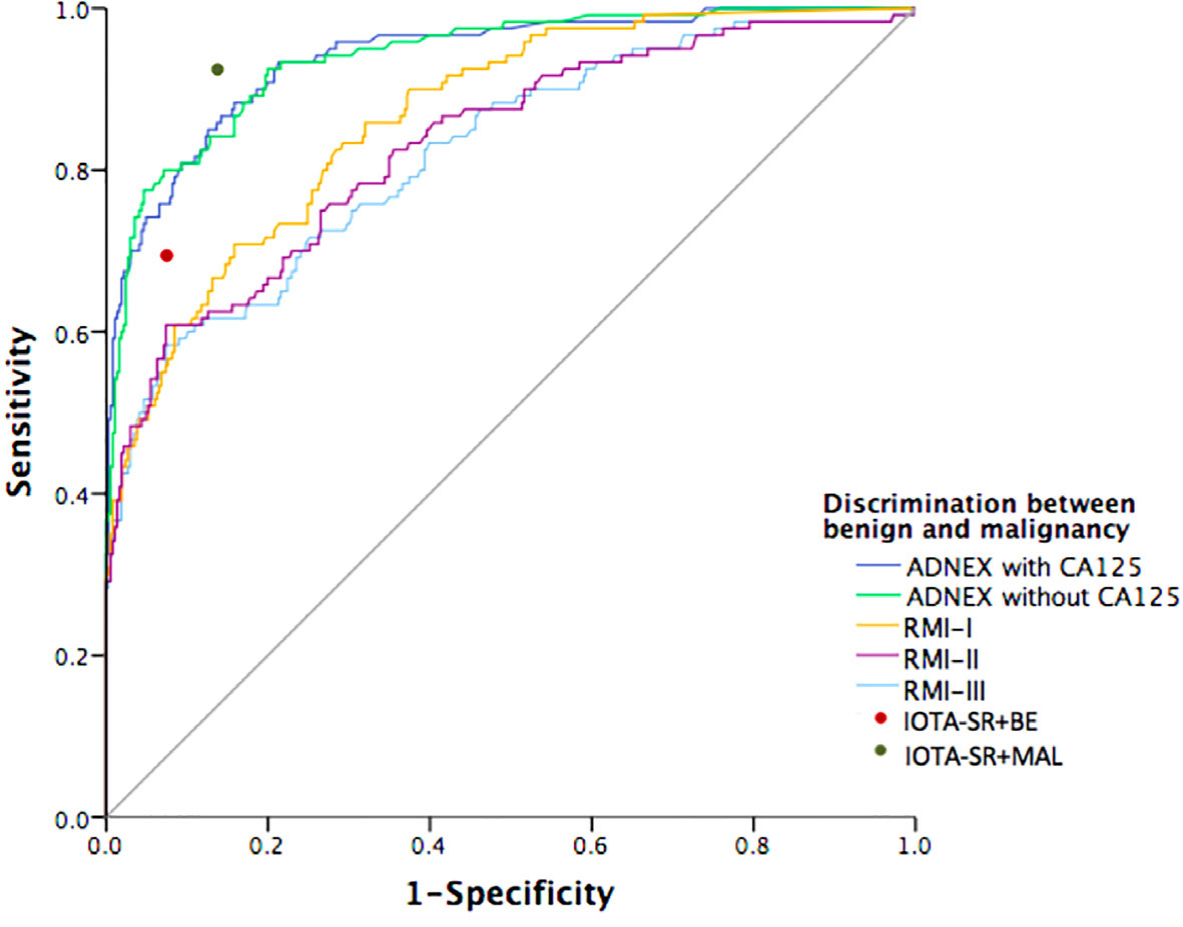
Receiver-operating characteristic curves for the performance of prediction models in detection of malignant adnexal masses. The prediction models including: Assessment of Different NEoplasias in the adneXa (ADNEX) model and three variants of the Risk of Malignancy Index (RMI). The red and green ROC point represents the Simple Rules model applied with inconclusive diagnoses classified as either benign (red) or malignant (green).

**Figure 2** shows the ROC curves for the ADNEX model and the RMI variants for differentiating malignant and benign tumors. The ADNEX model with or without CA125 was superior to the RMI variants regarding the diagnosis of malignant and benign tumors. When the SRs model yielded inconclusive results that were classified as malignancies, the model’s diagnostic performance was good.

**Table 4** summarises the pairwise ROC curve comparisons of the ADNEX model with or without CA125 and the RMI, which are expressed as differences in the AUCs. The difference between the AUCs for the ADNEX model with or without CA125 was not significant (AUC difference: 0.0002; 95% CI: 0.01–0.02). Comparisons of the ADNEX model with or without CA125 and the three RMI variants revealed significant differences in the AUCs that ranged from 0.074 to 0.118 (all P < 0.0001). Comparisons of the ADNEX model with and without CA125 with RMI variant I showed the greatest differences in the AUC (AUC difference: 0.074; 95% CIs: 0.039–0.109 and 0.040–0.108, respectively; P < 0.0001). The diagnostic performances of the three RMI variants remained statistically significant for the pre-operative diagnosis of adnexal masses (AUC differences: 0.010–0.044; all P < 0.05).

**Table 4:** Pairwise ROC curve comparisons expressed as differences in AUC and P-values for the study.

d-AUC (P*)	ADNEX^N125^	RMI-I	RMI-II	RMI-III
ADNEX^125^	0.0002 (-0.012-0.012) P=0.977	0.074 (0.039-0.109) P<0.0001	0.109 (0.066-0.151) P<0.0001	0.118 (0.076-0.160) P<0.0001
ADNEX^N125^	/	0.074 (0.040-0.108) P<0.0001	0.108 (0.064-0.153) P<0.0001	0.118 (0.073-0.163) P<0.0001
RMI-I	/	/	0.035 (0.010-0.060) P=0.007	0.044 (0.017-0.071) P=0.001
RMI-II	/	/	/	0.010 (0.003-0.016) P=0.002

*Comparisons of area under the curves (AUCs) of prediction models using Delong’s test; methods in left column are used as reference standard for comparisons; d-AUC, differences in area under the curve. Prediction models; ADNEX^125^, the Assessment of Different NEoplasias in the adneXa model with CA125 level; ADNEX^N125^, the Assessment of Different NEoplasias in the adneXa model without CA125 level; RMI-I, RMI-II, RMI-III, three variants of the Risk of Malignancy Index. Values in parentheses are 95% CI.

## Discussion

Correctly discriminating between benign and malignant adnexal masses is a crucial starting point for optimal treatment. We compared the diagnostic performances of the ADNEX and SRs models, and the RMI. The RMI was the first prediction model used clinically, and it is the most widely used model in many regions (4, 35, 36). However, our study’s findings showed that the ADNEX model was superior to the three RMI variants at distinguishing between benign and malignant adnexal masses. The ADNEX model with and without CA125 had higher AUCs (both 0.94) than the AUCs generated for the RMI variants that ranged from 0.82 to 0.87. Like previous studies’ findings (6, 28), the ADNEX model showed a better diagnostic performance and a higher level of sensitivity than the RMI in our study. Hence, the ADNEX model might be favoured for pre-operatively differentiating adnexal masses in Chinese patients.

Pre-operative evaluations using the SRs model were robust, with a sensitivity of 0.93 (95% CI: 0.86–0.97) and a specificity of 0.86 (95% CI: 0.82–0.89) for adnexal masses with inconclusive diagnoses that were classified as malignant; these findings are similar to the results from previous studies (6, 26, 30, 37, 38). The IOTA SRs model is widely accepted as an effective prediction model for adnexal masses by clinicians, and its use is recommended in the 2011 Green-top guidelines for the assessment and management of suspected ovarian masses in pre-menopausal women that were developed by the Royal College of Obstetricians and Gynaecologists in the United Kingdom (39). Recently, the American College of Obstetricians and Gynecologists incorporated the SRs model into their clinical practice guidelines for the evaluation and management of adnexal masses (40). Followed in the First International Consensus on Adnexal Masses the SRs model was recommended as the main diagnostic strategy (41). The SRs model is easy to apply in clinical practice, and it can be used for approximately 76-89% of adnexal masses (26, 42). The SRs model was applicable to about 86.8% of the patients in our study. When specialists in gynaecological ultrasonography are not available, classifying tumors as malignant is reasonable following inconclusive diagnoses using the SRs model (26, 43). However, this approach could be biased by the prevalence of malignant tumors within the population, and approximately half of the patients with benign diagnoses might undergo unnecessary interventions (26, 43).

Our analyses determined that 64 patients had tumors with inconclusive diagnoses following the application of the SRs protocol to the ADNEX model with or without CA125 and the three RMI variants. Compared with the three RMI variants, the AUC for the ADNEX model was higher (0.59 vs 0.73), the sensitivity was greater (0.29–0.36 vs 0.89), and the specificities were lower (0.86–0.89 vs 0.33–0.39) (**Supplementary Tables 1**, **2**). Regarding the tumors with inconclusive diagnoses, the prediction models’ AUCs did not differ, which may be attributable to the limited sample size. Nevertheless, regarding the identification of malignant tumors among the masses with inconclusive diagnoses, the ADNEX model yielded slightly higher AUCs and DORs than the three RMI variants.

This is one of the first studies to compare the ultrasound-based IOTA prediction models and the RMI in a population of Chinese patients in strict accordance with the IOTA consensus statement, which is a study strength. Additionally, we prospectively and consecutively enrolled unselected patients, and only patients whose data were complete were included. Moreover, our results were validated within a relatively large total study population between benign and malignant patients, however the sample size in particular subtypes was still limited. The study’s weakness, namely, its single-centre design, may have caused a sampling bias and limited the applicability of the results to other regions. Moreover, the ultrasound examinations were not performed by those with different levels of training experience in our study. More studies in different diagnostic centres with different levels of ultrasound expertise in China are needed to further evaluate the prediction models.

In conclusion, our study’s findings showed that the ADNEX and SRs models performed well in relation to discriminating between benign and malignant adnexal masses, and that both models were superior to the RMI in a Chinese context.

## Data Availability Statement

The raw data supporting the conclusions of this article will be made available by the authors, without undue reservation.

## Ethics Statement

The study was approved by the Ruijin Hospital, Shanghai Jiaotong University School of Medicine institutional ethics (Grant No.2018-136).

## Author Contributions

LQ: project development, data collection, data analysis and manuscript writing. QD: data analysis and manuscript editing. MJ: data collection and manuscript writing. FY: provided advice for the manuscript. HC: protocol and project development, appraised and revised the manuscript. WF: appraised and revised the manuscript. All authors contributed to the article and approved the submitted version.

## Funding

This work was sponsored by Medical Innovation Project of Shanghai Science and Technology Commission (20Y11914000), The ultrasonic model in diagnosis of ovarian tumors (KY20200033), Shanghai Sailing Program (18YF1414400) and National Natural Science Foundation of China (81901488).

## Conflict of Interest

The authors declare that the research was conducted in the absence of any commercial or financial relationships that could be construed as a potential conflict of interest.

## Abbreviations

ADNEX, Assessment of Different NEoplasias in the adnexa; AUC, area under the curve; BOT, borderline ovarian tumor; CA, carbohydrate antigen; Cis, confidence intervals; DOR, diagnostic odds ratio; FIGO, International Federation of Gynecology and Obstetrics; IOTA, International Ovarian Tumor Analysis; LR2, Logistic Regression model 2; MHz, megahertz; OC, ovarian cancer; ROC, receiver-operating characteristics; RMI, Risk of Malignancy Index; SRs, the Simple Rules; WHO, World Health Organization.
