# Supplementary material for: Comparison of the Diagnostic Performances of Ultrasound-Based Models for Predicting Malignancy in Patients With Adnexal Masses
Source: Front Oncol. 2021 Jun 1;11:673722. doi: 10.3389/fonc.2021.673722 (PMC8204044; doi:10.3389/fonc.2021.673722)
Supplement: Supplementary file 1 [file DataSheet_1.docx]

**Tables**

**STable 1** Diagnostic measurement results for strategies in identification of malignancy when SRs model yielded inconclusive diagnoses.

| *Strategy* | *AUC* | *Sensitivity* | *Specificity* | *PPV* | *NPV* | *LR+* | *LR-* | *DOR* |
| --- | --- | --- | --- | --- | --- | --- | --- | --- |
| ADNEX^125^ | 0.73  (0.60-0.83) | 0.89  (0.72-0.98) | 0.39  （0.23-0.57） | 0.59  (0.44-0.73) | 0.78  (0.51-0.95) | 1.46  (1.11-2.00) | 0.28  (0.09-0.90) | 5.21 |
| ADNEX^N125^ | 0.73  (0.61-0.84) | 0.89  (0.72-0.98) | 0.33  （0.19-0.51） | 0.57  (0.43-0.71) | 0.76  (0.46-0.94) | 1.34  (1.00-1.70) | 0.32  (0.10-1.00) | 4.19 |
| RMI-I | 0.59  (0.46-0.71) | 0.29  (0.13-0.49) | 0.86  （0.71-0.95） | 0.67  (0.38-0.89) | 0.55  (0.40-0.69) | 2.06  (0.80-5.60) | 0.83  (0.60-1.10) | 2.48 |
| RMI-II | 0.59  (0.46-0.71) | 0.36  (0.19-0.56) | 0.86  （0.71-0.95） | 0.72  (0.44-0.91) | 0.57  (0.42-0.71) | 2.57  (1.00-6.70) | 0.75  (0.50-1.00) | 3.43 |
| RMI-III | 0.59  (0.46-0.71) | 0.29  (0.13-0.49) | 0.89  （0.74-0.97） | 0.72  (0.41-0.93) | 0.55  (0.41-0.69) | 2.57  (0.90-7.70) | 0.80  (0.60-1.00) | 3.21 |
| Cases with inconclusive results in SRs model (n=64) | | | | | | | | |

Values in parentheses are 95% CI.Strategies: ADNEX^125^, the Assessment of Different NEoplasias in the adneXa model with CA125 level; ADNEX^N125^, the Assessment of Different NEoplasias in the adneXa model without CA125 level; RMI-I, RMI-II, RMI-III, three variants of the Risk of Malignancy Index. For ADNEX models, cut-off value of 10% was used and for the three variants of RMI model, cut-off value of 200 was used. AUC, area under receiver-operating characteristic curve; PPV, positive predictive value; NPV, negative predictive value; LR+, positive likelihood ratio; LR–, negative likelihood ratio; DOR, diagnostic odds ratio. Of the 13.2% (64/486) cases considered as inconclusive results in SRs model, 7.4% (36/486) benign tumors, 2.7% (13/486) borderline ovarian tumors, 1.9% (9/486) stage I ovarian cancer, 0.6% (3/486) stages II-IV ovarian cancer and 0.6% (3/486) ovarian metastases.

**STable 2** Pairwise ROC curve comparisons for strategies identification of malignancy when SRs model conceived inconclusive results.

| *d-AUC (P*)* | *ADNEX^N125^* | *RMI-I* | *RMI-II* | *RMI-III* |
| --- | --- | --- | --- | --- |
| ADNEX^125^ | 0.0049 (-0.046-0.056)  P=0.849 | 0.138 (-0.021-0.297)  P=0.089 | 0.139 (-0.019-0.297)  P=0.085 | 0.142 (-0.016-0.300)  P=0.079 |
| ADNEX^N125^ | / | 0.143 (-0.027-0.313)  P=0.100 | 0.144 (-0.031-0.319)  P=0.107 | 0.147 (-0.029-0.323)  P=0.102 |
| RMI-I | / | / | 0.0010 (-0.075-0.077)  P=0.980 | 0.0040 (-0.076-0.084)  P=0.923 |
| RMI-II | / | / | / | 0.0030 (-0.016-0.022)  P=0.762 |

* Comparisons expressed as differences in area under the curves (AUCs) of prediction models using Delong’s test; methods in left column are used as reference standard for comparisons; d-AUC, differences in area under the curve. Prediction model strategies: ADNEX^125^, the Assessment of Different NEoplasias in the adneXa model with CA125 level; ADNEX^N125^, the Assessment of Different NEoplasias in the adneXa model without CA125 level; RMI-I, RMI-II, RMI-III, three variants of the Risk of Malignancy Index. Values in parentheses are 95% CI.
